# Assessing *MC1R* Variants in Lentigo Maligna Melanoma within the Utah Population

**DOI:** 10.1158/2767-9764.CRC-25-0263

**Published:** 2025-07-28

**Authors:** Amanda Jiang, Annabelle Huntsman, Carly Becker, Bing-Jian Feng, Kayla Marks, Jessica Donigan, Keith L. Duffy, Alice Frigerio, Douglas Grossman, Deborah W. Neklason, Robert L. Judson-Torres, Dekker C. Deacon

**Affiliations:** 1 Huntsman Cancer Institute, Salt Lake City, Utah.; 2 Department of Oncological Sciences, Huntsman Cancer Institute, University of Utah, Salt Lake City, Utah.; 3 School of Medicine, University of Utah, Salt Lake City, Utah.; 4 Department of Dermatology, University of Utah, Salt Lake City, Utah.; 5 Division of Epidemiology, Department of Internal Medicine, University of Utah, Salt Lake City, Utah.

## Abstract

Lentigo maligna (LM) and lentigo maligna melanoma (LMM) arise from chronically sun-damaged skin. LM/LMM incidence continues to increase, particularly in Utah, where melanoma rates are twice the national average. The melanocortin-1 receptor (*MC1R*) has been studied in melanocyte pigmentation and DNA repair but has yet to be thoroughly investigated in LM/LMM. We investigated allele and genotype frequencies of germline *MC1R* variants among 175 Utah patients diagnosed with LM/LMM and 402 Utah reference individuals. The comparative analysis demonstrated an increased frequency of the D294H allele (0.046; *P* = 0.0042) and a decreased frequency of the V60L allele (0.074; *P* = 0.034) in patients with LM/LMM. The LM/LMM group demonstrated a higher OR compared with the Utah reference group associated with R151C homozygosity compared with heterozygous R151C [OR = 5.6; 95% confidence interval (CI), 0.98–32; *P* = 0.052] and R151C homozygosity compared with wild type (OR = 5.7; 95% CI, 1.1–30; *P* = 0.042). D294H heterozygosity was strongly associated with LM/LMM (OR = 3.8; 95% CI, 1.3–11; *P* = 0.014). Conversely, V60L heterozygosity was less strongly associated with LM/LMM (OR = 0.52; 95% CI, 0.26–1.1; *P* = 0.072). Stratified analyses showed no significant differences in age or gender across the key *MC1R* variants studied. These data highlight significant differences in *MC1R* allele frequencies in patients with LM/LMM, demonstrating that D294H is associated with increased LM/LMM risk, whereas the V60L variant is inversely associated with risk. This study provides the first comprehensive analysis of specific high-risk *MC1R* variants in patients with LM/LMM in Utah.

**Significance:**

Our study is the first comprehensive analysis of *MC1R* germline variants in patients with LM/LMM in Utah, a region with an exceptionally high melanoma incidence. We draw new risk associations in LM/LMM, identifying an increased risk with the D294H and R151C variants. We also describe a novel inverse association for V60L, warranting further investigation. This study contributes to improved targeted risk stratification and an increased understanding of an understudied melanoma subtype.

## Introduction

Cutaneous melanomas (CM) are broadly classified based on the presence or absence of chronic sun-induced damage. CM associated with chronic sun-induced damage includes lentigo maligna (LM), nodular melanoma, and desmoplastic melanoma, which typically manifest on the head, neck, or dorsal surfaces of the extremities (1). Despite comprising 4% to 15% of all CMs (2), the genetic predisposition underlying the development and progression of LM remains inadequately understood. A review of melanomas diagnosed over a 10-year span in our department revealed a disproportionate increase in LM cases, which accounted for the majority of observed increases in CM diagnoses (3). LM, an *in situ* form of CM, poses challenges for identification due to its slow growth and similar appearance to other pigmented skin lesions. When LM transitions to an invasive state, lentigo maligna melanoma (LMM), survival rates significantly decline. The prognosis for stage III and IV LMM is similarly poor compared with other CM subtypes, including acral lentiginous melanoma and nodular melanoma (4). Advancing our understanding of the genetic risk factors that affect the development of LM/LMM may enhance prognostic accuracy and risk stratification.

The melanocortin-1 receptor (*MC1R*) gene has been studied in pigmentation regulation and, more recently, in DNA repair mechanisms. The *MC1R* gene encodes seven transmembrane G protein–coupled receptors that exhibit a strong affinity for α–melanocyte-stimulating hormone, initiating downstream pathways that result in brown/black eumelanin synthesis (5). Conversely, impaired *MC1R* signaling results in increased red/yellow pheomelanin synthesis, which has been shown to provide weak shielding capacity against UV radiation (UVR) and can amplify reactive oxygen species (6). Consequently, an increased ratio of eumelanin to pheomelanin is considered protective against UVR-induced damage and in scavenging free radicals (7).

Among the nine most prevalent high-risk germline *MC1R* variants, “R” variants (D84E, R142H, R151C, I155T, R160W, and D294H) are associated with the most significant CM risk and are strongly linked to the red hair phenotype, whereas “r” variants (V60L, V92M, and R163Q) exhibit weaker associations with CM risk (8). Previous research indicates that the risk of CM is increased 2.2-fold in individuals carrying one R variant and 4.1-fold in those carrying two R variants (9), with the most strongly associated variants being R151C, R160W, and V60L (10). These studies did not differentiate between CM subtypes, resulting in bias toward the most common types of CM (i.e., superficial spreading melanoma) with limited examination of patients with LM/LMM subtypes. One study breaking down the CM subtypes identified a significant association between the R163Q variant and LMM development (OR: 2.2, *P* value = 0.044), independent of phenotypic features of fair skin and red hair, in 166 patients with LMM from Spain (11). The limited studies on the allelic frequency of *MC1R* variants in patients who develop LM/LMM represent a significant knowledge gap, particularly in regions with higher CM prevalence and individuals with chronic sun damage. Utah exhibits a higher prevalence of CM and a predominately Northern European ancestry, both of which contribute to an increased risk for sun-related skin cancers. The high Northern European ancestry is related to a large founding Mormon population. Although there is a constant influx of new populations and inbreeding rates are low, the frequency of some genetic alleles can be different from broad cohorts of European ancestry (12, 13). Melanoma ranks as the third most prevalent malignancy in Utah, with incidence rates nearly twice those of the rest of the country (14). Implementing more specific guidelines for screening can enhance CM detection rates and reduce associated mortality (15).

Our study focuses on genetic predisposition and assessing the frequency of high-risk *MC1R* variants in the Utah LM/LMM population. However, we acknowledge that LM/LMM development is multifactorial, with established risk factors, including age and gender. Although our study was not designed to evaluate all the potential risk factors, our demographic analyses showed no significant differences in age or gender across these *MC1R* variants, suggesting that these demographic factors are unlikely to confound our key findings. Identifying the prevalence of various *MC1R* variants in LM/LMM within the Utah population may yield additional insights into the pathogenesis of melanoma subtype development and may inform strategies for skin cancer screening in higher-risk patient populations.

## Materials and Methods

### Sample collection and DNA extraction

Discarded tissues were collected at the Huntsman Cancer Hospital from Mohs surgeries done for patients diagnosed with LM and LMM under umbrella protocol 00076927, project ID #90, approved by the University of Utah Institutional Review Board. Nontumor samples that were discarded after standard clinical care were taken from cases with and without residual disease. Inclusion criteria included discarded samples from patients diagnosed with LM/LMM who underwent and provided consent for Mohs surgery. Individuals with other types of melanomas or skin conditions were excluded from the study. As this study did not involve human participants, written informed consent was not required. All study procedures were done in accordance with the Declaration of Helsinki. A total of 230 discarded samples were collected, 55 of which we were unable to extract DNA from or Sanger sequence for results. This was an attrition rate of 23.9%. Subject demographics are provided in Table 1. DNA was extracted from ≤25 mg of tissue per sample using the QIAamp DNA Mini Kit (QIAGEN, Cat. #51304) and eluted in 10 mmol/L Tris/HCl (pH 8.0). The extracted DNA was quantified using the Qubit dsDNA Quantitation, High Sensitivity assay (Thermo Fisher Scientific). PCR to amplify the *MC1R* locus was performed using 150 ng of genomic DNA input and following the Takara PrimeSTAR Max DNA Polymerase protocol (Takara, Cat. #R045A v201510Da) as previously described (16). PCR products were size confirmed with gel electrophoresis using a 1% agarose gel. DNA bands were removed from the gel and underwent the NucleoSpin Gel and PCR Clean-up kit protocol (MACHEREY-NAGEL, Cat. #740609.50).

**Table 1:** Key demographic characteristics of patients with LM/LMM in the study (*n* = 175)

Characteristic	
Total patients	175
Gender	​
Male	69.14%
Female	30.86%
Age	​
Median	73
Mean	71.46
Minimum	36
Maximum	98
Ethnicity	​
Non-Hispanic	93.14%
Hispanic	0.57%
N/A^a^	6.29%
Location	​
Head	85.14%
Trunk	7.43%
Extremities	7.43%
LM/LMM	​
LM	78.86%
LMM	21.14%

a N/A refers to patients for whom self-reported ethnicity data were not collected.

### Sanger sequencing

Two separate Sanger sequencing reactions were performed for each sample: one with the *MC1R* forward primer and another with the *MC1R* reverse primer, to cover all variants of interest. Clean-up genomic DNA (150 ng) was used in each Sanger sequencing reaction. The obtained sequences were analyzed using ApE (17), and the quality was assessed based on the chromatogram confidence bars. The sequences were aligned with a wild-type (WT) sequence, and any mutations were noted.

### Single Nucleotide Polymorphism Database *MC1R* variant allele frequencies

The allele frequency data for *MC1R* variants were obtained from the Single Nucleotide Polymorphism Database (dbSNP) (RRID: SCR 002338; ref. 18), a public archive for simple nucleotide polymorphisms and multiple small-scale variations. Each *MC1R* variant was identified using the dbSNP search function, and data for prevalence in different populations were extracted. Allele frequency data were retrieved for African, European, Asian, and other relevant population groups and compiled, including variant ID (rs number) and frequencies. Some population subgroups were combined: The African group consisted of African, African other, and African American; the Asian subgroup was made up of Asian, East Asian, South Asian, and other Asian; Latin American 1 and Latin American 2 were combined into Latin American. Allele frequency was calculated as the proportion of alternative alleles to reference alleles.

### Utah reference group

Genomic data for the Utah reference group were obtained from the 1000 Genome Project (RRID: SCR 006828), the Utah Centre d’Etudes du Polymorphisme Humain project (Database of Genotypes and Phenotypes study: phs001872.v1, Genome Sequencing of Large, Multigenerational Centre d’Etudes du Polymorphisme Humain/Utah Families), and a collection of noncancer studies that underwent whole-genome sequencing (Heritage 1K project). Broad use of these appropriately consented samples was granted as use did not meet the federal definition of human subjects research as determined by the University of Utah Institutional Review Board (#0085908). Unrelated Utah-specific research participants were selected, resulting in a reference group of 402 patients. The allele frequencies in this Utah reference group were compared with those from our patient population with LM/LMM.

### Statistical analysis

Statistical analyses were performed to compare the allele frequency of various *MC1R* variants in the LM population with a reference Utah population. Differences in scores were assessed using the Mann–Whitney U test. Samples were all independent of one another, and the tests were run with a nonparametric assumption. A *P* value < 0.05 was considered statistically significant. All statistical analyses were performed using Prism version 10.2.1 (GraphPad Software). The ORs for specific genotypes were calculated by comparing the ratios of specific *MC1R* genotype combinations between the LM/LMM cohort and the Utah reference group. This produced an OR for which we calculated a *P* value and confidence intervals.

### Data availability

The complete dataset, including all allele frequencies and statistical analyses, is available upon request. The Sanger sequencing files can be found in Supplementary Data file S1.

dbSNP entries for the *MC1R* variants analyzed can be accessed using the following rs numbers. Data were accessed in February 2025.

rs1805006 (D84E)

rs11547464 (R142H)

rs1805007 (R151C)

rs1110400 (I155T)

rs1805008 (R160W)

rs1805009 (D294H)

rs1805005 (V60L)

rs2228479 (V92M)

rs885479 (R163Q)

## Results

### Demographic characteristics

We assembled a cohort of 175 patients referred for Mohs micrographic surgery for the treatment of LM or LMM (Table 1). Within this cohort, 69.14% of participants were male, and 30.86% were female, which correlates with the established observation that the lifetime risk of CM is higher in males (19). The median age of the patients was 73 years. The majority of patients (93.14%) self-identified as non-Hispanic White. Anatomic distribution analysis demonstrated that the head was the most common site of occurrence, accounting for 85.14% of cases, followed by the extremities (7.43%) and the trunk (7.43%).

To assess whether age or gender may confound the observed associations between *MC1R* variants and the patients with LM/LMM, we performed additional analyses comparing these demographic variables across the *MC1R* variants of interest. There were no significant differences in age or gender distribution across variant groups (Supplementary Fig. S1). These findings suggest that neither age nor gender is likely to account for the variant-specific associations observed in our cohort.

### Comparison of *MC1R* variant allele frequencies in the overall population, compared with patients with LM/LMM and a Utah reference group

**Figure 1:**
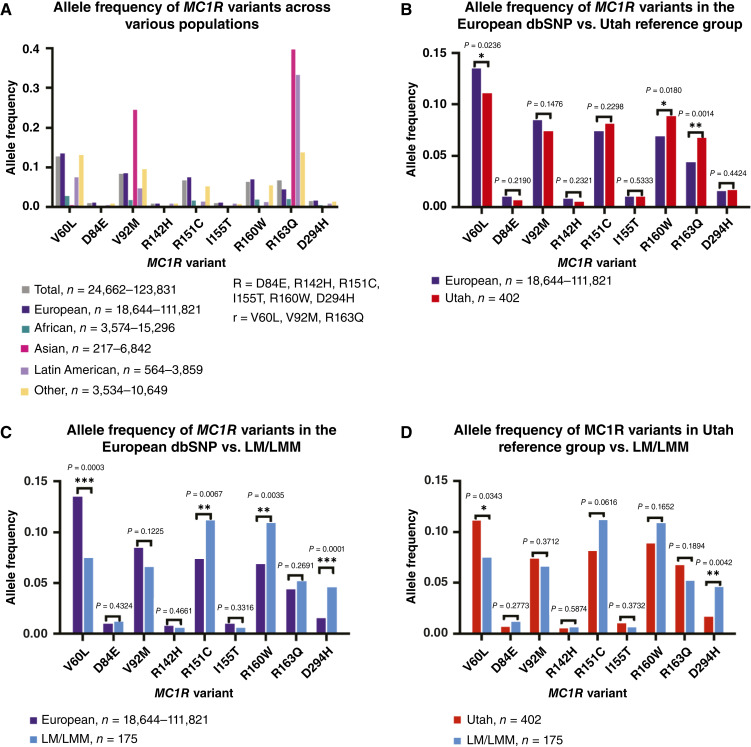
*MC1R* variant allele frequencies vary between the Utah reference group and LM/LMM cohorts. **A,** Bar graph of *MC1R* allele frequencies in different populations derived from the dSNP database, *N* = 24,662–123,831. **B,** Bar graph comparing the European dbSNP data (*N* = 18,644–111,821) with the Utah reference group (*N* = 402). **C,** Bar graph comparing the European dbSNP data (*N* = 18,644–111,821) with the LM/LMM cohort (*N* = 175). **D,** Bar graph comparing the *MC1R* allele frequencies between the LM/LMM cohort (*N* = 175) and the Utah reference group (*N* = 402). Significance in **B–D** was assessed using Fisher’s exact test, with a *P* value < 0.05 considered statistically significant. *P* value significance thresholds: <0.05 (*), <0.01 (**), and <0.001 (***).

We compiled germline *MC1R* variant frequency data from different ethnic populations using the dbSNP (Fig. 1A). Individuals of Latin American ancestry had an increased frequency of the R163Q variant, whereas individuals of Asian ancestry had an increased frequency of both R163Q and V92M. In contrast, among those of European ancestry, the V60L, R151C, and R160W variants had higher frequencies. In all populations, there were low allele frequencies for the D84E, R142H, I155T, and D294H variants. We compared the dbSNP data with our Utah reference group and our Mohs LM/LMM cohort to investigate population-level differences in allele frequencies. Given that the majority of patients in our cohorts identified as non-Hispanic White, we used only the European ancestry dbSNP data to make the most accurate comparisons. According to the 2020 census, 75.4% of Utah’s population identified as White (Utah 2020 Census), and 93.14% of our cohort of patients with LM/LMM identified as non-Hispanic White (Table 1).

We initially compared the European dbSNP data with the Utah reference group of 402 genetically unrelated individuals from Utah (Fig. 1B). This cohort served as a reference group rather than a control group due to the unknown melanoma status of those included. In the Utah reference group, the allele frequency of V60L (*P* = 0.024) was significantly lower compared with the European population. Additionally, the frequencies of the R160W (*P* = 0.018) and R163Q (*P* = 0.0014) alleles were higher in the Utah reference group compared with the European dbSNP data.

Next, we evaluated the differences in *MC1R* variant frequencies between our Mohs LM/LMM cohort and the European dbSNP population (Fig. 1C). In the Mohs patients with LM/LMM, there was a significant decrease in the V60L variant frequency (*P* = 0.0003). Conversely, in the Mohs LM/LMM cohort, the R151C (*P* = 0.0067), R160W (*P* = 0.0035), and D294H (*P* = 0.0001) variants demonstrated increased prevalence.

Comparison of the Utah reference group and the Mohs patients highlighted significant differences in *MC1R* allele variant frequency (Fig. 1D). Notably, in the Mohs LM/LMM group, the frequency of the D294H “R” variant was significantly increased (0.046; *P* = 0.0042) compared with the Utah reference group (0.016). In contrast, in the LM/LMM cohort, the V60L “r” variant had a significantly lower allele frequency prevalence compared with the Utah reference group (LM/LMM = 0.074, Utah = 0.11, *P* = 0.034). The other “r” variants, V92M and R163Q, had similar allele frequencies between the LM/LMM cohort and the Utah reference group. In the cohort of patients with LM/LMM, the R151C “R” allele was trending higher compared with the Utah reference group (LM/LMM = 0.11, Utah = 0.081, *P* = 0.062). All raw data underlying the analyses, including individual genotypes and clinical features, are provided in Supplementary Tables S1–S3.

### Genotype frequency analysis

**Figure 2:**
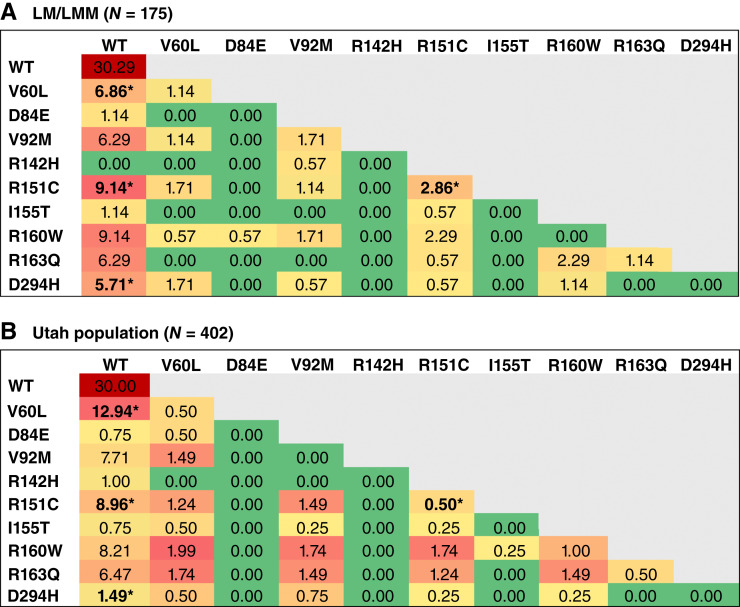
Specific *MC1R* genotypes confer an increased risk of developing LM/LMM. The heatmaps display the percentage frequencies of *MC1R* genotypes within two cohorts: (**A**) Mohs LM/LMM cohort and (**B**) the Utah reference group. The heatmaps are color-coded to reflect genotype percentages, with red indicating higher frequencies and green indicating lower frequencies. This comparison highlights the differential distribution of *MC1R* genotypes between patients with LM/LMM and the Utah reference group. * denotes samples for which we calculated an OR compared with WT or heterozygous/homozygous genotypes.

Finally, we compared the prevalence of *MC1R* variant and WT genotypes of our Mohs patients with LM/LMM to those of the Utah reference group (Fig. 2) to determine the CM risk associated with each genotype. The OR was calculated by comparing the number of samples with a particular genotype in the LM/LMM cohort and Utah reference group to the WT genotype frequency in each cohort. In the Mohs LM/LMM cohort, 6.82% of patients were heterozygous for the V60L/WT genotype, compared with 12.94% in the Utah reference group. The OR of developing LM/LMM in patients with V60L/WT relative to WT was lower compared with the Utah reference group [OR = 0.52; 95% confidence interval (CI), 0.26–1.1; *P* = 0.072]. In contrast, 2.84% of the LM/LMM cohort were homozygous for the R151C variant, compared with 0.5% in the Utah reference group. Patients with LM/LMM were nearly six times more likely to be homozygous for R151C compared with those with a WT genotype (OR = 5.7; 95% CI, 1.1–30; *P* = 0.042). Furthermore, when compared with patients with an R151C/WT heterozygous genotype, patients with LM/LMM were 5.6 times more likely to be homozygous for R151C (OR = 5.6; 95% CI, 0.98–32; *P* = 0.052). The D294H/WT heterozygous genotype was associated with developing LM/LMM, with our LM/LMM cohort having a higher OR (OR = 3.8; 95% CI, 1.3–11; *P* = 0.014) compared with WT patients. Collectively, these data demonstrate that R151C homozygosity or heterozygosity and D294H heterozygosity are associated with an increased likelihood of developing LM/LMM. Interestingly, V60L heterozygosity was less prevalent among the Mohs patients with LM/LMM than in the Utah reference group (OR = 0.52; 95% CI, 0.26–1.1; *P* = 0.072).

## Discussion

The presence of germline *MC1R* allele variants has been associated with the risk of developing CM, yet this association has not been well characterized in patients with the LM/LMM subtype. This study investigated the association of “R” and “r” *MC1R* variants in LM/LMM within the Utah population. The Utah population is particularly interesting due to the notably high incidence of CM cases, primarily attributed to an increasing prevalence of LM/LMM (3). Given that CM rates are also influenced by heightened diagnostic scrutiny and lower clinical thresholds for biopsy (20), screening efforts should prioritize patients with factors that increase susceptibility to CM, thereby refining the scope of preventative measures. Our findings highlight significant differences in *MC1R* allele frequencies associated with LM/LMM. To our knowledge, this is the first study evaluating the prevalence of *MC1R* variants in Utah patients with LM/LMM.

*MC1R* gene variants predispose patients to CM through impaired responses to oxidative stress and diminished DNA repair mechanisms (21, 22). *In vivo* studies using murine models with premature termination of the *MC1R* transcript have demonstrated accelerated melanoma development due to oxidative damage resulting from an increased pheomelanin-to-eumelanin ratio, even in the absence of UVR exposure (23). In the context of LM/LMM subtypes, which typically arise on chronically sun-damaged skin, understanding the interplay between *MC1R* variants and UVR-independent mechanisms may improve patient risk stratification and screening recommendations based on *MC1R* genotypes. However, a study utilizing *MC1R* status as a proxy for CM risk in Utah found that the capacity of the antioxidant N-acetylcysteine to protect nevi against UVR-induced oxidative stress was not affected by *MC1R* genotype (16).

Our analyses demonstrated considerable variability in the frequency of *MC1R* variants across populations. The comparisons between the dbSNP data, a Utah reference group, and patients with LM/LMM are important because this CM subtype is predominantly diagnosed in patients self-identifying as White (24). Consistent with prior findings, the “R” variants R151C and R160W were more prevalent in European populations (9). Our patients with LM/LMM had a higher frequency of the R151C and R160W alleles, consistent with the associations of these variants with higher CM risk (10). A positive correlation was observed between the R151C allele and our Mohs LM/LMM cohort, but this was not statistically significant. Power calculations for a Fisher’s exact test revealed that our sample size was underpowered, requiring at least 476 Mohs LM/LMM samples to achieve 80% power at a 0.05 significance level. Additional statistically significant associations might be identified with a larger sample size and increased power.

In the Utah reference group, we saw a significantly increased frequency of the R160W and R163Q variants compared with the European dbSNP data, suggesting that these could be predisposing risk factors for the elevated CM rates in Utah. In the LM/LMM cohort, we identified no significant increase in the R163Q variant compared with the Utah reference group or the European dbSNP data. This finding is in contrast with the only other study looking at *MC1R* variants in Mediterranean patients with LM (11). This discrepancy of R163Q not being significantly associated with LM/LMM in our cohort may be attributed to the predominantly Northern European ancestry of the Utah population in this study, highlighting the differential effects of *MC1R* variants based on ethnicity. This analysis highlights a population-specific risk for CM, as different variants contribute varying levels of risk across local and global populations.

We observed a significantly higher prevalence of the D294H “R” variant in our Mohs patients with LM/LMM compared with the Utah reference group, along with a trending increase in R151C allele frequency in our Mohs LM/LMM cohort. These findings emphasize the potential role of D294H and R151C in developing LM/LMM, supporting previous studies that link “R” variants to an increased risk of melanoma (6, 10, 11, 25, 26). Specifically, R151C homozygosity was significantly associated with an increased risk for LM/LMM. Conversely, the V60L “r” variant exhibited a lower frequency in patients with LM/LMM compared with the Utah reference group and the European dbSNP data, suggesting a possible protective effect against these melanoma subtypes. In prior studies, the V60L variant had the fourth highest attributable risk percentage in nonmelanoma skin cancer (27). In studies of sporadic CM of unspecified subtypes, V60L posed the third highest risk (10). Sporadic CM typically arises on intermittently sun-exposed skin, whereas LM/LMMs develop on chronically sun-damaged areas. The V60L *MC1R* variant may increase susceptibility to UVR-induced DNA damage in melanocytes on intermittently sun-exposed skin, whereas in chronically sun-damaged skin, in which LM/LMMs arise, cumulative UVR exposure and mutations in other pathways (TP53, NRAS, TERT, etc.) may play a more significant role. Thus, in chronically sun-damaged skin, the V60L mutation would render a protective effect in LM/LMM. However, the exact mechanism of this putative protective effect remains to be elucidated.

In a prior study of non-subtyped CM genotypes, CM risk was increased with a V60L (OR = 1.3), R151C (OR = 2.1), R160W (OR = 1.9), or D294H (OR = 2.3) allele (9). Similarly, in our study, we saw an increased risk associated with R151C and D294H. Conversely, in our study, V60L heterozygous patients with LM/LMM had a decreased risk of LM/LMM. A study of LMM in Mediterranean patients showed a significant association of the R163Q allele (OR = 2.2; *P* = 0.044) with CM risk. We did not see this in our specific population of patients with LM/LMM, and this discrepancy may result from differences in ethnicity frequencies within the datasets, emphasizing population-level differences.

Our study has several limitations. First, the relatively small sample size for patients with LM/LMM limited the statistical power for certain comparisons such as the association between the R151C allele and LM/LMM status. Second, the predominantly Northern European ancestry of Utah limits the generalizability of this study to more diverse populations. Lastly, an important limitation is the lack of adjustment for known LM/LMM risk factors, such as UV exposure history, age, and gender. Although we were not able to obtain clinical information about exposure to UV, we conducted analyses to assess age and gender. These analyses showed no significant differences in age or gender distribution across the *MC1R* variants studied. The gender distribution observed in our LM/LMM cohort, with a higher proportion of male patients, is consistent with established epidemiologic patterns for LM/LMM, which are more commonly diagnosed in older males with chronic sun exposure. In future work, we aim to build a more comprehensive multivariable risk model that can incorporate age and gender with a larger sample size. The novelty of this research is that this is the first study in Utah to identify the association between different *MC1R* variants and the development of LM/LMM. We have developed a well-characterized cohort of 175 patients with LM/LMM, a subtype of melanoma that is typically underrepresented in broader melanoma research yet is increasing in prevalence (3), providing insight into population-specific risk factors. In addition, we highlight the differential impact of *MC1R* genotypes, demonstrating that homozygosity for R151C and heterozygosity for D294H increased the risk for LM/LMM.

In conclusion, these data highlight significant differences in *MC1R* allele frequencies between patients with LM/LMM and the general Utah population, identifying D294H as being associated with increased LM/LMM risk, whereas the V60L variant should be evaluated in a larger cohort to validate the suggestive protective effect. These findings highlight the importance of a deeper understanding of the genetics of different CM subtypes. Further research is warranted to explore the mechanisms underlying the protective effects of specific *MC1R* variants and to validate our findings in larger, more diverse populations.

## Supplementary Material

Supplementary Figure 1 Figure of age and gender distribution for key MC1R variants.

Supplementary Table 1 Table of dbSNP allele frequencies

Supplementary Table 2 Table of the Utah reference group data

Supplementary Table 3 Table of data in the Utah LM/LMM cohort

Supplemental Data Sanger sequencing data for each sample
