# Supplementary material for: Assessing MC1R Variants in Lentigo Maligna Melanoma within the Utah Population
Source: Cancer Res Commun. 2025 Jul 28;5(7):1228–34. doi: 10.1158/2767-9764.CRC-25-0263 (PMC12301710; doi:10.1158/2767-9764.CRC-25-0263)

Supplemental Figure 1. Age and gender distribution across *MC1R* variant groups in LM/LMM patients. A) Scatter plot of the age distribution of LM/LMM patients in our cohort. A Tukey's multiple comparisons test identified no significant differences when comparing each of the groups. B) Bar graph displaying the number of female and male patients within each *MC1R* variant category. A chi-squared test of independence indicated no significant difference between then female proportions and the male proportions among the groups ($\chi^2 = 2.32$, degrees of freedom = 4, $p = 0.678$).

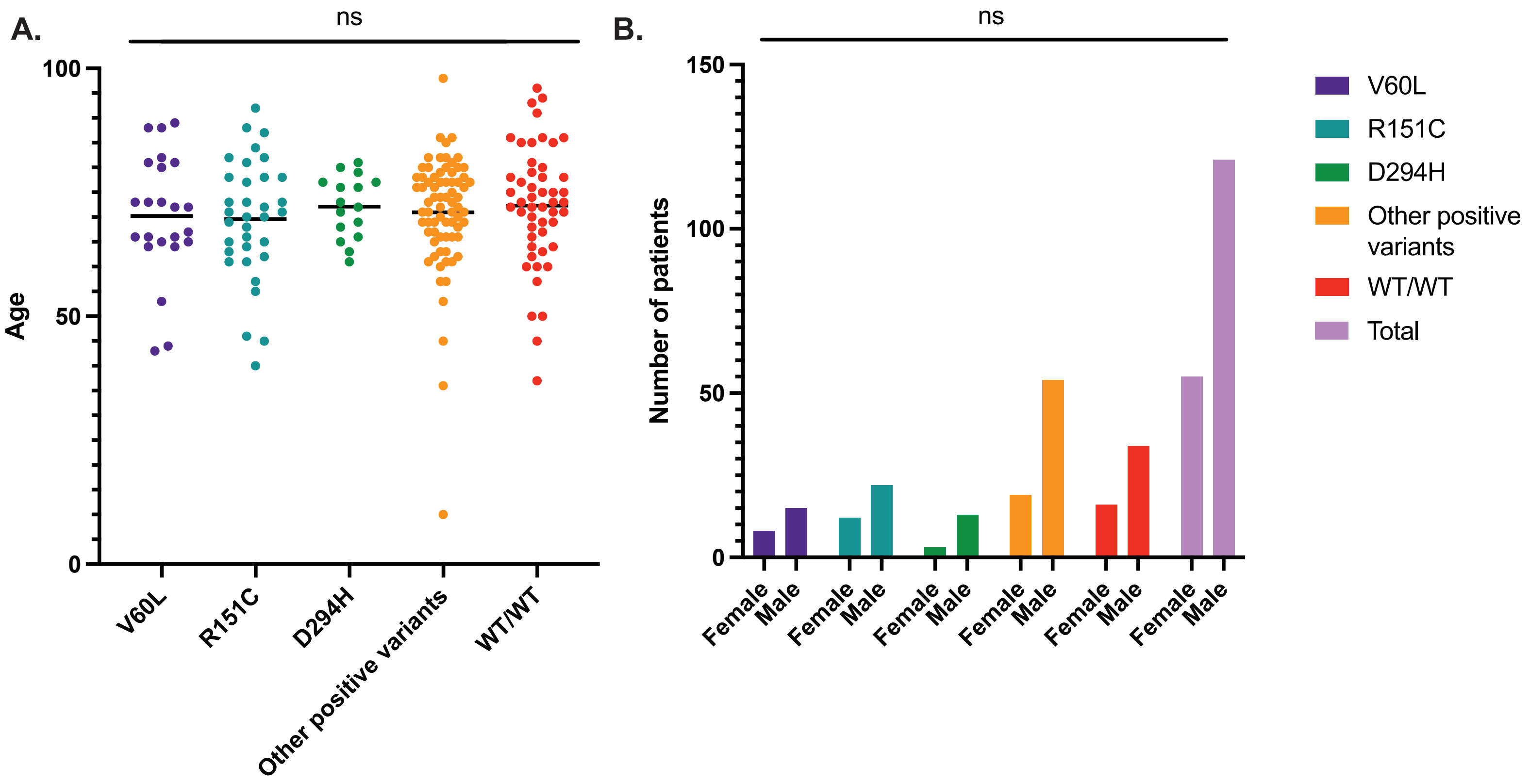

Supplement: Supplementary Figure 1 — Figure of age and gender distribution for key MC1R variants. [file crc-25-0263_supplementary_figure_1_suppsf1.pdf]
