# Supplementary material for: Assessing MC1R Variants in Lentigo Maligna Melanoma within the Utah Population
Source: Cancer Res Commun. 2025 Jul 28;5(7):1228–34. doi: 10.1158/2767-9764.CRC-25-0263 (PMC12301710; doi:10.1158/2767-9764.CRC-25-0263)
Supplement: Supplemental Data — Sanger sequencing data for each sample [file crc-25-0263_supplemental_data_suppsd.zip › SS files/README.rtf]

This is a collection of the sanger sequencing data 
